# Determinants of female condom use among female tertiary students in the Hohoe Municipality of Ghana using the Health Belief Model

**DOI:** 10.4314/ahs.v22i1.2

**Published:** 2022-03

**Authors:** Enyonam Amevor, Elvis Tarkang

**Affiliations:** 1 Department of population and behavioural sciences, school of public health, university of health and allied sciences PMB 31 Ho, Ghana; 2 HIV/AIDS prevention research network Cameroon, P. O. Box 36 Kumba, Cameroon

**Keywords:** Female condom use, Health Belief Model, Female tertiary students, Hohoe, Ghana

## Abstract

**Background:** Besides abstinence, the condom has proven to be the only effective method of preventing sexually transmitted infections (STIs), including HIV/AIDS. This study investigated the determinants of female condom (FC) use among female tertiary students in the Hohoe Municipality, Ghana using the Health Belief Model (HBM). Methods: A cross-sectional design was adopted. Data were collected using a structured questionnaire in January 2019 and analysed using STATA version 14.0. Logistic regression was used to measure the strength of associations between the dependent and independent variables. A p-value <0.05 was considered statistically significant. Results: The overall utilisation of the FC was 35.0%. Among the constructs of the HBM, it was perceived self-efficacy for FC use that was significantly associated with FC use: respondents who had the confidence to convince their partners to use the FC were 2 times more likely to use it than respondents who did not [AOR =2.15(CI: 1.26, 3.71); p= 0.005]. Conclusion: Female students in the current study exhibited poor utilization of the FC. Health promotion interventions should, therefore, focus on increasing their self-efficacy for FC use.

## Background

Adolescent females who indulge in unprotected sexual intercourse are at high risk of contracting sexually transmitted infections (STIs) including HIV/AIDS1. Globally, HIV/AIDS continue to be the leading cause of death among women of childbearing age (15–49 years) and the second leading cause of death for young women aged 15–24 years in Africa^2^. However, condom remains the only protection against the sexual transmission of HIV among sexually active adolescents^2^.

Physiologic susceptibility and gender inequalities put girls and young women at a higher risk of contracting HIV/AIDS than their male counterparts of the same age group^2^. The female condom (FC) is controlled by the woman and it can be inserted several hours before sexual intercourse; when used correctly, it is estimated to be 94–97% effective in reducing the risk of HIV transmission^3^. Therefore, providing the FC as a comprehensive HIV prevention strategy could result in increased levels of protection.

Despite the efficacy of the FC in preventing HIV transmission, relatively low utilization rates are still reported^4^,^5^. The determinants of FC use among female tertiary students could be of utmost importance for public health promoters in planning effective FC-related programmes, especially in high HIV prevalence settings like Hohoe, Ghana. The HIV prevalence in Hohoe stands at 3.4%, which is above the national prevalence of 1.5%^6^. Most tertiary students in sub-Saharan Africa (SSA) including Hohoe, engage in unprotected sexual intercourse^7^. Therefore, there is a high likelihood of encountering an adolescent or young adult who is HIV-positive. This may account for the relatively high HIV prevalence in Hohoe.

Every successful HIV prevention programme is based on theories about how and why people change their behaviours. The Health Belief Model (HBM) was developed to address this issue. This paper uses the perception constructs of the HBM, namely perceived susceptibility, perceived severity, perceived benefit, perceived barriers and perceived self-efficacy, as the conceptual framework to examine the determinants of female condom use among female tertiary students in the Hohoe Municipality of Ghana.

The HBM is built on the premise that:

- a person will take a health-related action (female condom use) if she believes that a negative health condition (HIV) can be avoided
- a person will avoid a negative health condition (HIV) if she has a positive expectation of the recommended action (female condom use)
- a person will take a health-related action if she believes that she can successfully take the recommended health action (the use of the female condom comfortably and with confidence)^8^,^9^.

No study in Ghana has investigated the determinants of FC use among female tertiary students using the HBM as the conceptual framework.

The HBM has been applied to various health promotion behaviours such as osteoporosis prevention^10^,^11^; prevention of heart failure^12^–^14^ obesity and overweight^15^; HIV risk perception^16^ and condom use^17^. Thus, justifying its use in the current study determine the psychosocial factors influencing contraceptive use among adolescent mothers in the Volta Region of Ghana.

It was hypothesized in this study that female tertiary students who perceive themselves susceptible to HIV transmission, perceive HIV/AIDS as a serious infection, perceive the female condom to be effective in HIV/AIDS prevention, perceive fewer barriers to using the female condom and believe in their ability to successfully use the female condom will be more likely to use it during sexual intercourse^9^.

## Methods

### Study site description

From the data gathered from the 2010 Ghana Population and Housing Census^18^, Hohoe Municipality has a population of 167,016 inhabitants, which represents 7.9% of the total population of the Volta Region. This consists of 52.1% females and 47.9% males, with children and adolescents under 15 years constituting 35.9% of the population. The republic of Togo borders the Municipality to the east, while to the west is the Kpando District. To the northwest is the Jasikan District and to the south is the Ho Municipal. The Municipality is made up of four tertiary institutions namely: The University of Health and Allied Sciences, Midwifery Training College, Saint Francis College of Education and Saint Theresa's College of Education. The student's population in these four institutions can be approximated to about 3,000.

### Study population

The accessible population included all female students who were studying in the four tertiary institutions in the Hohoe Municipality of Ghana; that portion of the target population to which the researcher had reasonable access.

### Inclusion criteria

The inclusion criteria were female students who were present and consented to participate in the study. For those below 18 years of age, parental/guardian consent was obtained while the participants gave assent.

### Exclusion criteria

Students who were severely ill or were absent from school on the day of data collection were excluded from the study.

### Study design

A quantitative cross-sectional design was adopted and data were collected in January 2019.

### Sample size determination

The sample size for the current study was 398. This was obtained by using Raosoft online sample size calculation, with a total population of 2,300, a margin of error of 0.05, Z score of 1.96 at 95% Confidence Interval (CI), an estimated proportion of FC use among female students of 50.0%. and 0.2 (20%) non-response rate (because of the sensitive nature of the study). Therefore, a total of 398 students who met the study inclusion criteria were recruited to take part in the study.

### Sampling method

Stratified sampling method was employed to select the students from all the four tertiary institutions within the Hohoe Municipality. This sampling method was used to group the students into 4 strata based on their level of study (100 level, 200 level, 300 level and 400 level). The list of the students was used as the sampling frame. The number of studentsor each stratum was comparably apportioned and a simple random sampling technique was used to select the samples. This was attained by writing the matching numbers against the names of the students on pieces of papers and placing them into a box and then selecting the sample without replacement based on the determined sample size.

### Data collection procedure

Data were collected using a structured self-administered anonymous questionnaire adapted from previous studies^16^,^17^, during a normal class period in January 2019. The questionnaire was pretested on a convenience sample of 20 female students who did not take part in the actual study, for clarity and to ascertain internal consistency.

Respondents were given the self-administered questionnaires in English, which was their first language. The students were closely supervised by two trained female research assistants of the same age group as the respondents, while they completed the questionnaires. The completed questionnaires were checked by the research assistants for errors and missing data before participants were allowed to go. Anonymously completed questionnaires were kept in a separate container from the signed informed consent forms to maintain anonymity.

### Measures

#### Outcome (Dependent) variable: female condom use

The outcome variable of interest for the current study was having ever used a female condom during sexual intercourse as reported by the female students. The question asked sexually active respondents: “Have you ever used a female condom during sexual intercourse?” The response options were categorized into ‘1=yes' and '0=no’.

### Explanatory (Independent) variables: constructs of the HBM

- Perceived susceptibility to HIV: This was measured with the item ‘I am at high risk of contracting HIV’. The response options were categorized into ‘1=yes’ and ‘0=no’.
- Perceived severity of HIV/AIDS: This was measured with the following statement: ‘The consequences of acquiring HIV are so serious’. The response options were categorized into ‘1=yes’ and ‘0=no’.
- The perceived benefit of female condom use: This was measured with the following statement: ‘Consistent use of the female condom prevents HIV transmission’. The response options were categorized into ‘1=yes’ and ‘0=no’.
- Perceived barriers to female condom use: This was measured with the following statements considered separately: ‘Female condom is very expensive’ and ‘Female condom use reduces sexual pleasure’. The response options were categorized into ‘1=yes’ and ‘0=no’.
- Perceived self-efficacy for female condom use: This was measured with the following statements considered separately: ‘I have the confidence to convince my partner to use the female condom during sexual intercourse’ and ‘I have the confidence to refuse sex with my partner if he refuses that I should use the female condom’. The response options were categorized into ‘1=yes’ and ‘0=no’.

### Data analysis

Data collected from the study were entered into Epi Data 3.1 and exported into STATA 14.0 for cleaning and analysis. Binomial logistic regression was performed to examine the probability of using the FC during sexual intercourse. Binomial logistic regression predicts the probability that an observation falls into one of two categories of a dichotomous dependent variable (female condom use) based on one or more independent variables that can be either continuous or categorical (constructs of the HBM).

For binomial logistic regression to be performed, the following assumptions must be met:

- The dependent variable should be measured on a dichotomous scale
- One or more independent variables which can be either continuous (interval or ratio variable) or categorical (ordinal or nominal)
- Independence of observations and the dependent variable should have mutually exclusive and exhaustive categories^19^.

The procedure gives rise to estimates of odds of a certain event occurring (use of the female condom), given a set of explanatory variables (the constructs of the HBM).

All these assumptions were fulfilled in the current study, thus justifying the use of binomial logistic regression to investigate the determinants of the female condom among female tertiary students in the Hohoe municipality, Ghana.

To estimate the odds ratios (OR), all the components of the HBM and the socio-demographic variables were entered into the first model to evaluate possible interactions. Significant interaction terms were retained and entered in a general model. The sequence of covariate removal from the model was determined by the likelihood ratio testing and the Hosmer-Lemeshow goodness-of-fit test to ensure that the covariate that contributed the least to the fit of the model would be removed first while adjusting for the simultaneous effects of other sets of variables in the model. The significance level for all statistical tests was 5%.

### Ethical issues

Ethical approval to conduct this study was obtained from the Research Ethics Committee of the University of Health and Allied Sciences (UHAS-REC A.4 [4] 18–19). This study was conducted following all accepted principles on the ethics of this REC. Permission was obtained from the administrative authorities of each school before the commencement of data collection. Informed consent was obtained from participants on standard consent form before their inclusion in the study. For those below 18 years of age, parental consent was obtained while they gave assent to participate in the study. The purpose of the study was fully disclosed to the participants and their consent was obtained after disclosure. Students were informed that their participation was voluntary and they were assured of confidentiality and anonymity, that under no circumstance will their names and other details be linked to the data analysis and dissemination of findings of the study.

## Results

### Socio-demographic characteristics of the respondents

Table 1 shows the demographic characteristics of the respondents. A total of 398 female respondents were surveyed with a mean age of 25.2±19.4. The majority, 248 (62.3%) were aged 20–29 years; most, 376(94.7%) were Christians; the majority, 259(65.1%) were Ewes; 195(49.0%) were in their first year of study; 315(79.1%) were single; 119(30.0) were from TERESCO; most of their fathers, 202(50.7%) had a tertiary level of education and majority of their mothers, 121(30.4%) also had a tertiary level of education.

**Table 1:** Socio-demographic characteristics of the respondents

Variable	Frequency [N=398]	Percentage (%)
**Mean age in years (S.D)**	25.2±19.4	
**Age (Years)**		
<20	71	18.0
20–29	248	62.2
30–39	73	18.3
40+	6	1.5
**Religion**		
Christian	376	94.7
Muslim	21	5.3
**Ethnicity**		
Ewe	259	65.1
Akan	75	18.8
Guan	27	6.8
Fanti	14	3.5
Other	23	5.8
**Year of study**		
First year	195	49.0
Second year	120	30.1
Third year	48	12.1
Fourth year	35	8.8
**Marital status**		
Single	315	79.1
Married	82	20.6
Other	1	0.3
**Institution of study**		
UHAS	106	26.6
FRANCO	59	14.8
MIDWIFERY	114	28.6
TERESCO	119	30.0
**Father's level of education**		
No formal education	38	9.6
Primary	10	2.5
JHS	65	16.3
SHS	83	20.9
Tertiary	202	50.7
**Mother's level of education**		
No formal education	53	13.3
Primary	37	9.3
JHS	112	28.2
SHS	75	18.8
Tertiary	121	30.4

JHS: Junior High School; SHS: Senior High School

### Utilization of the female condom

Of the 398 respondents, 331 (83.2%) had experienced sexual intercourse and 116 (35.0%) had used the female condom.

### Determinants of female condom based on the constructs of the HBM

Table 2 describes determinants of FC use. Of the respondents, majority 244(61.5%) believed they were not at a high risk of contracting HIV (perceived susceptibility), even though the majority, 337(84.9%) knew that the consequences of acquiring HIV are serious (perceived severity); most, 366(92.2%) believed that using the FC consistently would prevent HIV transmission (perceived benefit); the majority, 342 (86.2%) believed that the FC is not very costly and most, 275(69.3%) claimed the FC oes not reduces sexual pleasure (perceived barriers); the majority, 280(70.5%) had the confidence to convince their partners to use the FC and 213(53.6%) had the confidence to refuse sex if their partners refused to use the FC (perceived self-efficacy).

**Table 2:** Determinants of female condom use based on the constructs of the HBM

Variable	Frequency [N=398]	Percentage (%)
**Perceived susceptibility**		
***Perception of risk of contracting HIV***		
At high risk	153	38.5
Not at risk	244	61.5
**Perceived severity**		
***Perceived seriousness of consequences of acquiring HIV***		
Serious	337	84.9
Not serious	60	15.1
**Perceived benefit**		
***The perceived benefit of the female condom in preventing HIV***		
Beneficial	366	92.2
Not beneficial	31	7.8
**The perceived barrier to female condom use**		
***Perceived cost of the female condom***		
Expensive	55	13.9
Not expensive	342	86.2
***Perceived reduction of sexual pleasure by female condom***		
Reduced pleasure	122	30.7
Not reduced	275	69.3
**Perceived self-efficacy for female condom use**		
***Perceived confidence to convince my partner to use the female*** ***condom***		
Confidence	280	70.5
No confidence	117	29.5
***Perceived confidence to refuse sex with my partner if he refuses*** ***female condom use***		
Confidence	213	53.6
No confidence	184	46.4

Table 3 shows the association between sociodemographic characteristics, the constructs of the HBM and the utilization of the FC. Respondents who could convince their partners to use the FC during sexual intercourse (perceived self-efficacy) were 2 times more likely to use the FC than those who lacked the confidence [AOR = 2.15(C. I: 1.26, 3.71) p= 0.005].

**Table 3:** Association between demographic characteristics, constructs of the HBM and utilisation of female condom

	Utilisation of the FC		Chi-square		
Variable	No n(%)	Yes n(%)	(p-value)	COR(95%C.I)p-value	AOR(95%C.I)p-value
**Age (Years)**					
<20	47(16.7)	24(20.7)		Ref	
20–29	184(65.3)	64(55.2)		0.68(0.39, 1.20) 0.185	
30–39	46(16.3)	27(23.3)		1.15(0.58, 2.28) 0.690	
40+	5(1.7)	1(0.9)	4.71(0.194)	0.39(0.04, 3.54) 0.404	
**Religion**					
Christian	267(94.7)	110(94.8)		Ref	
Muslim	15(5.3)	6(5.2)	0.00(0.953)	0.97(0.37, 2.57) 0.953	
**Ethnicity**					
Ewe	189(67.0)	70(60.3)		Ref	
Akan	51(18.1)	24(20.7)		1.27(0.73,0.73) 0.400	
Guan	19(6.7)	8(6.9)		1.14(.48, 2.71) 0.773	
Fanti	6(2.1)	8(6.9)		3.60(1.21, 10.74) 0.022	
Other	17(6.0)	6(5.2)	6.28(0.179)	0.95(.36, 2.51) 0.922	
**Year**					
First-year	136(48.2)	59(50.9)		Ref	
Second-Year	88(31.2)	32(27.6)		0.84(0.50, 1.39) 0.495	
Third-Year	34(12.1)	14(12.1)		0.95(0.47, 1.90) 0.883	
Fourth-Year	24(8.5)	11(9.5)	0.56(0.905)	1.06(0.47, 1.90) 0.883	
**Marital status**					
Single	226(80.1)	89(76.7)		Ref	
Married	56(19.9)	27(23.3)	0.58(0.446)	1.22(0.73, 2.06) 0.446	
Other					
**Institution of study**					
UHAS	62(22.0)	44(37.9)		2.28(1.12, 4.66,) 0.976	
FRANCO	45(16.0)	14(12.1)			
MIDWIFERY	84(29.8)	30(25.9)		1.15(0.55, 2.38) 0.711	
TERESCO	91(32.3)	28(24.1)	**10.94(0.012)**	0.99(0.47, 2.06) 0.976	
**Father's level of education**					
No formal education	28(9.9)	10(8.6)		Ref	
Primary	7(2.5)	3(2.6)		1.2(0.26, 5.55) 0.816	
J.H.S	50(17.7)	15(12.9)		0.84(0.33,2.12) 0.712	
S.H.S	55(19.5)	28(24.1)		1.43(0.61, 3.35) 0.416	
Tertiary	142(50.4)	60(51.7)	2.18(0.701)	1.18(0.54, 2.59) 0.674	
**Mother's level of education**					
No formal education	42(14.9)	11(9.5)		Ref	
Primary	25(8.90	12 (10.3)		1.83(0.70, 4.77) 0.214	
J.H.S	80(28.4)	32(27.6)		1.53(0.70, 3.33) 0.287	
S.H.S	52(18.4)	23(19.8)		1.69(0.74, 3.86) 0.213	
Tertiary	83(29.43)	38(32.8)	2.40(0.662)	1.75(0.81, 3.76) 0.153	
**Perceived susceptibility**					
***Perceived risk of contracting HIV***					
Not at risk	177(62.8)	67(57.8)		Ref	
At risk	105(37.2)	49(42.2)	0.86(0.351)	1.23(0.79, 1.92) 0.352	
**Perceived severity**					
***Perceived seriousness of consequences*** ***of acquiring HIV***					
Not serious	41(14.5)	19(16.4)		Ref	
Serious	241(85.5)	97(83.6)	0.21(0.641)	0.87(0.48, 1.57) 0.641	
**Perceived benefit**					
***The perceived benefit of the female*** ***condom in preventing HIV***					
Not beneficial	24(8.5)	7(6.0)		Ref	
Beneficial	258(91.5)	109(94.0)	0.70(0.402)	1.45(0.61, 3.46) 0.405	
**Perceived barrier**					
***Perceived cost of the female condom***					
Not expensive	241(85.5)	101(87.1)		Ref	
Expensive	41(14.5)	15(12.9)	0.17(0.675)	0.87(0.46, 1.65) 0.675	
***Perceived reduction of sexual pleasure*** ***by female condom***					
Not reduced	189(67.0)	86(74.1)		Ref	
Pleasure reduced	93(33.0)	30(25.9)	1.94(0.163)	0.71(0.44, 1.15) 0.164	
**Perceived Self-efficacy**					
***Perceived confidence to convince my*** ***partner to use the female condom***					
No confidence	97(34.4)	21(18.1)		Ref	
Confidence	185(65.6)	95(81.9)	**10.46(0.001)**	**2.37(1.39, 4.04) 0.001**	**2.15(1.26, 3.71) 0.005**
***Perceived confidence to refuse sex with*** ***my partner if he refuses to use the*** ***female condom***					
Confidence	146(51.8)	68(58.6)		1.32(0.85, 2.04) 0.214	
No confidence	136(48.2)	48(41.4)	1.55(0.213)		

## Discussion

The HBM proposes that a student will take a health preventive action (FC use) if she regards herself as susceptible to a health condition (HIV/AIDS), believes that the recommended action available to her (FC use) would be beneficial in reducing her susceptible if she believes that the anticipated barriers to taking the recommended action are outweighed by the benefits^8^,^9^.

The current study revealed that although female tertiary students exhibited low perceived susceptibility to HIV, their perception of the severity of HIV/AIDS and the benefit of the FC were high and their perceived barriers to FC use was quite low. However, their perceived self-efficacy for FC use was quite high (Table 2). These results translated to 35% utilisation of the FC during sexual intercourse among the respondents who had experienced sex.

In affirmation, an intervention study in South Africa among female university students showed that only 4.1% of participants reported having ever used the FC at baseline, prior to the intervention. At the first follow up, 62.6% reported having used the FC post-intervention. However, at the second follow up, 38.2% reported that they had used the FC since their first follow up interview^20^.

In the current study, the majority (61.5%) of the students did not perceive themselves to be at a high risk of contracting HIV/AIDS (perceived susceptibility), and therefore, they might not see the need of using the FC during sexual intercourse to prevent the disease. The reason for the low-risk perception among the students could be that since they are tertiary-level students they might downplay their risk perception with the view that their sexual behaviours may not put them at risk of contracting HIV. Additionally, since HIV/AIDS is a highly stigmatised disease, acknowledging their risk of contracting the disease implies putting themselves at risk of being stigmatised^21^. There is, therefore, the need for health promotion programmes to heighten the perception of risk of contracting HIV among female tertiary students in Hohoe, Ghana, so that they may see the need to use the FC during sexual intercourse to prevent HIV transmission.

Female students who perceived themselves to be at a high risk of contracting HIV, may not be motivated to use the FC during sexual intercourse unless they realise that contracting HIV may pose severe consequences to them (perceived severity). It is when they realise the magnitude of the negative consequences of HIV, that they would take the necessary actions (using FC consistently) to prevent HIV transmission^9^. In the current study, the 15.1% of students who did not perceive that acquiring HIV may pose serious consequences to them, may not see the need of using the FC during sexual intercourse to prevent its transmission. These finding emphasises the necessity of strategies to increase the perception of the severity of HIV/AIDS among the students.

From Table 2, majority of the students (92.2%) perceived that FC use is beneficial in preventing HIV transmission. This perception could motivate them to use FC during sexual intercourse. Female condoms are 94–97% effective in preventing HIV transmission when used correctly^3^.

Any obstacles to the use of the FC can interfere with its usage during sexual intercourse to prevent HIV transmission^22^. Perceived barriers refer to one's belief in the tangible and psychological costs of the advised behaviours against a condition or problem^23^. Students in the current study perceived some barriers to using the FC during sexual intercourse.

These students may find it very difficult to use the FC during sexual intercourse to prevent HIV transmission. Discussing FC use with partner and believing one's partner holds positive attitudes toward the FC can also affect its use during sexual intercourse^20^. In South Africa, at the end of an intervention on FC, some women still failed to convince male partners to use the FC, often due to its physical attributes or partners' lack of knowledge about insertion^24^. There is a need for health promotion interventions to empower these students with strategies to overcome such barriers so that they can use the FC consistently during sexual intercourse to prevent HIV transmission.

It is only when students realise that they can deal with these barriers, that they would be able to take the recommended actions. Perceived self-efficacy is the strength of a student's belief in her ability to respond to novel or difficult situations and to deal with any associated obstacles or setbacks^9^. A student should feel that she is capable of taking the necessary action correctly because it is that confidence that would motivate her to initiate and sustain the action. Results from the current study reveal that 29.2% of the students did not have the confidence that they could convince their partners to use the FC during sexual intercourse. This is in contrast to findings from a study conducted in Southwestern Nigeria among tertiary students, which reported that less than half of the participants (44.6%) felt bold/confident enough to use a female condom during sexual intercourse^25^.

Also, in the current study, 46.4% did not have the confidence that they could refuse sexual intercourse with their partners if they refused to use the FC (perceived self-efficacy). This corresponds with a study conducted among tertiary students in Southwestern Nigeria, which revealed that about one-third (35.1%) attested that persuading their partners to accept the utilization of a female condom will be difficult based on health (STI/HIV) reasons while 43.6% participants disagree with the perception^25^. The FC empowers women to protect themselves from both STIs and unintended pregnancy, but targeted interventions are needed to address men's negative attitudes toward the device^20^.

According to the HBM, female students who perceive themselves to be at risk of contracting HIV/AIDS, need to have the confidence that they can use the FC before they could use it to prevent HIV transmission. Students with low self-efficacy for FC use may not use it during sexual intercourse to prevent HIV transmission. This was reflected in the current study as only 35.0% of the students reported FC usage during sex.

In the current study, female students who had the confidence to convince their partners to use the FC were more likely to use it during sexual intercourse to prevent HIV transmission. This finding is consistent with those of other studies conducted in Zimbabwe^26^ and Northern California^27^, which report perceived self-efficacy for FC use as a significant determinant of its usage. Further, a South African intervention on FC use showed that most women (30/39) applied information learned during the interventions to negotiate with partners. Also, the women reported that FC insertion practice increased their confidence^24^.

Based on the assumptions of the HBM^28^, we conclude that without female students' perception of being at high risk of contracting HIV (perceived susceptibility), there could be no resultant preventative actions (using the FC) against HIV transmission. Therefore, the perception of risk of contracting HIV is assumed to be the immediate antecedent of the use of the FC to prevent HIV transmission. Therefore, the lack of significant associations between the other components of the HBM and FC use could be the result of the low perception of risk of contracting HIV (perceived susceptibility) among the female students, which resulted in low use of the FC during sexual intercourse (29.2%).

The hypothesis that female tertiary students who perceive themselves susceptible to HIV transmission, who perceive HIV/AIDS as a serious infection, who perceive the FC to be effective in HIV/AIDS prevention, who perceive fewer barriers to using the FC and believe in their ability to successfully use the FC would be more likely to use it during sexual intercourse is partially accepted based on our analyses.

The current study points to the need for interventions to increase the perception of risk of contracting HIV among female students and strategies to empower them with FC negotiation skills. This study also suggests that AIDS prevention programmes among female tertiary students in Hohoe should implement strategies to help them to overcome tangible and psycho-social barriers to FC use. These strategies should also include information on how to use the female condom correctly. It must be emphasized that with practice, the female condom becomes easier and more fun to use. The FC can become a regular and pleasurable part of a romantic relationship. Furthermore, the government of Ghana should consider subsidizing the FC, to bring it within the financial reach of the young and poor female students, making them readily available in public health structures (hospitals and health centres).

## Limitations

First, the study used a self-reporting instrument (questionnaire) that has the potential of introducing social desirability bias and there was no way to validate what the respondents reported. However, the assurance of anonymity and confidentiality of the responses should have minimized possible misreporting. Second, this study was restricted to only four schools in the Volta Region. The findings are therefore not generalizable to other regions in the country. Third, the study was done in a predominantly Christian environment. Results could differ in Muslim settings.

## Conclusion

Female condom use among female tertiary students in Hohoe is low and is statistically associated with perceived self-efficacy for FC use. Health promotion interventions to improve FC use should empower the female students with FC negotiation skills to increase their self-efficacy for FC use.

## Data Availability

Data will be made available upon request.
